# Editorial: Nanomicrobiology: Emerging Trends in Microbial Synthesis of Nanomaterials and Their Applications

**DOI:** 10.3389/fmicb.2021.751693

**Published:** 2021-10-28

**Authors:** Sougata Ghosh, Raymond J. Turner

**Affiliations:** ^1^Department of Microbiology, School of Science, RK University, Rajkot, India; ^2^Department of Biological Sciences, University of Calgary, Calgary, AB, Canada

**Keywords:** nanostructures, microbial synthesis, toxicology, environmental bioremediation, therapeutic applications

The Research Topic entitled **“***Nanomicrobiology: Emerging Trends in Microbial Synthesis of Nanomaterials and their Applications***”** was dedicated to reviews and original research articles toward innovative environmentally benign routes for synthesis of nanoparticles using microbial metabolites as catalysts and stabilizing agents. Microbial nanofactories involve the several microbial metabolites that play a key role in the reduction of metal ions to form nanoparticles with exotic shape, size, physicochemical, and optoelectronic properties. Microbial polysaccharides, peptides, and secondary metabolites help in stabilization of the biogenic nanoparticles. Metal ions can interact with the carboxylic, amino, and sulfhydryl functional groups which further help in their reduction to metal nanoparticles (Rana et al., 2020). Microbial nanofactory derived cationic polysaccharides such as chitosan and anionic polymer like alginate can adhere to the surface of the nanoparticles. Similarly, dextran and pullulan are neutral polymer that can also help in capping or stabilization of the biogenic nanoparticles. Further, chitosan can form complex with DNA/RNA and hence can be used in drug/gene delivery systems (Mizrahy and Peer, 2012). Among several amino acids are responsible for the shape evolution of the AuNPs (Doyen et al., 2016).

Enzyme catalyzed metabolic routes play a significant role in synthesis of nanoparticles in microbes. Several low molecular weight peptide, glutathione (GSH), and proteins such as metallothioneins and phytochelatins are responsible for synthesis of nanoparticles. Similarly, enzymes such as oxidoreductases, NADH-dependent nitrate reductases (NRs), and cysteine desulfhydrases facilitate the biosynthesis of metallic nanostructures within the microbial cells (Rana et al., 2020).

Binding of secondary structures of proteins with the microbially synthesized nanoparticle can enhance biodegradability, impart high stability, significantly reduce toxicity and antigenicity (Bunschoten et al., 2012; Elzoghby et al., 2012).

Chemical and physical routes of nanoparticle synthesis often use toxic chemicals and hazardous reaction conditions. Thus, the articles published under this Research Topic cover microbes as preferred resources for synthesizing nanoparticles with attractive features and diverse applications. Chemolithotrophy mediated generation of energy, cellular integration, and detoxification are considered as key mechanisms for microbial synthesis of nanoparticles.

The review of Mandeep and Shukla highlighted the promising role of nanobiotechnological potential of microbes for bioremediation of industrial effluents. The ability of nanostructures associated with microbes such as *Pseudomonas aeruginosa, Lysinibacillus* sp., Actinomycetes are shown to remove toxic metals (chromium, copper, cobalt, nickel, zinc) along with hazardous dyes and antibiotics. This showcases the promising role of biogenic nanoparticles for ensuring clean water and safe environment toward the UN sustainability goals. Enzymes like reductases, metabolites such as peptides, aliphatic, and aromatic compounds help in metal ion reduction due to electron shuttle or capping of charge.

Biologically generated nanoparticles have immense applications which are highlighted in this special issue. Microbially synthesized silver, platinum, zinc oxide, copper oxide nanoparticles can have promising antibacterial, antifungal, and antiviral activity. Similarly, microbe assisted fabrication of gold nanoparticles may help to inhibit the cancer and reactive oxygen species mediated oxidative stress. Magnetic nanoparticles synthesized by bacteria can be used for targeting specific tissue for drug delivery using external magnetic field. Further, biogenic nanoparticles can be exploited for their uses in food packaging, agriculture, water treatment, and medicine.

Another significant application of the nanoparticles is detection, toxicity, and management of different types of mycotoxins. Nanotechnology driven solution to address the perils of mycotoxicology are not only rapid but also efficient, economical, and specific. This can help in disease management, disease forecasting, controlling disease resistance, detection of mycotoxin, and diagnosis of the toxicity (Rai and Abd-Elsalam, 2019).

Ranpariya et al. demonstrated the role the phytogenic bimetallic nanoparticles comprised of silver and platinum as novel antimicrobial agents. The nanohybrids with size ranging from 20 to 80 nm exhibited high antimicrobial activity against *P. aeruginosa* and *Staphylococcus aureus*. Interestingly, the biogenic nanoparticles showed promising antimicrobial synergy when used in combination with wide range of antibiotics. This can be a powerful strategy to control indiscriminate use of high dosage of antibiotics, which is one of the reasons for emerging multidrug resistance among bacterial pathogens.

In another review, Rattan et al. emphasized the role of lichens, such as *Parmotrema praesorediosum, Parmotrema clavuliferum, Parmelia perlata, Ramalina dumeticola* for producing nanoparticles with broad spectrum antimicrobial activity against both Gram-positive and Gram-negative bacterial pathogens. Further, they presented a comprehensive overview of potential mechanisms in understanding the mode of action of the biogenic nanoparticles against bacteria. Such mechanisms include damage to the cell wall and membranes for efficient penetration within the bacterial cell finally disintegrating the cellular components leading to impairment of the cellular metabolism leading to cell death. Another interesting article by Lahiri et al. elaborated on the biofilm inhibitory potential of the microbially synthesized nanoparticles. They explained that effective penetration of the nanoparticles through the biofilm matrix composed of exopolysaccharides is critical for affecting the quorum-sensing gene cascades that eventually hinders cell-to-cell communication resulting in inhibition of biofilm structure.

Dhanker et al. in their most elaborate and informative review have correlated the expression of metal resistance genes (MRG) in microbes with their ability to synthesize nanoparticles. An interdisciplinary approach using biotechnology, molecular biology, metabolic engineering, synthetic biology, and genetic engineering has led to the development of environmentally benign routes for synthesis of nanoparticles using diverse microbial communities that include bacteria (*Lactobacillus, Rhodopseudomonas capsulate, Rhodococcus* spp., *Shewanella algae*), fungi and yeasts (*Cryptococcus laurentii, Pichia pastoris, Rhodotorula glutinis*) protozoa (*Leishmania* sp., *Tetrahymena thermophila SB210, Tetrahymena pyriformis*), and archae (*Halobiforma* sp. N1, *Sulfolobus acidocaldarius, Sulfolobus tokadaii*). Similarly, Ghosh et al. presented a mechanistic approach for microbial synthesis of nanoparticles where nanotization was considered as a way to ameliorate stress in microbe. Moreover, they established the fact that biogenesis of nanoparticles is a biodefense mechanism in microbes that involves metal excretion/accumulation across membranes, enzymatic action, efflux pump systems, binding at peptides, and precipitation. Hence, exploration of the interaction between metal ions with proteins, DNA, organelles, membranes, and their subsequent cellular uptake would help in the deciphering the cellular, biochemical, and molecular mechanisms of nanotization of metals. *Delftia acidovorans* was reported to secrete a secondary metabolite and a non-ribosomal peptide “delftibactin” which reduced Au^3+^ into Au^0^ and adhered to them, thus reducing its toxicity.

Kapoor et al. indicated the advantages of microbial route for fabrication of nanoparticles. This easy, rapid, low cost, clean, non-toxic, environmentally benign, and sustainable approach for reducing metal into their corresponding nanoparticles followed by stabilization can serve as a pollution abatement tool. Hence, micro-organisms such as, bacteria, actinomycetes, filamentous fungi, yeast, algae, and viruses can led to bioremediation by eliminating toxic metals and promote environmental cleanup. On a similar note, Mukherjee et al. presented the promises of algae and blue green algae for synthesis of nanoparticles. They explored the possibilities of various applications of the phycogenic nanoparticles such as antioxidant, antibacterial, and antifungal activities. In another interesting study exploring the fungus *Beauveria bassiana* encapsulated in nanoparticles of biopolymers such as, soy oil, corn starch, cellulose, lignin, alginate, and humic acid showed better biocontrol properties against *Spodoptera cosmioides* with a mortality rate of up to 90% (Felizatti et al.). Hence such formulations can have promising applications for pest control that strongly rationalize the application of nanotechnology for agriculture.

Also, the enzyme hydrogenase was principally believed to be involved in reduction of U^6+^ and Se^6+^ by *Micrococcus lactyliticus* and *Clostridium pasteurianum*, respectively. Likewise, hydrogenases from sulfate reducing bacteria (SRB) is capable of reducing metals such as Tc^7+^ and Cr^6+^. Riddin et al. (2006) proposed the mechanism of reduction of Pt(IV) to Pt(0) in SRB (Sulfate Reducing Bacteria) in a two-step enzymatic process wherein Pt(IV) was first reduced to Pt(II), an intermediate ion by oxygen-sensitive cytoplasmic hydrogenase/reductant, which then diffused to periplasmic space where it gets reduced to Pt(0) by an oxygen-tolerant periplasmic hydrogenase enzyme.

The content of this Special Issue can be summarized as use of microbially synthesized diverse nanomaterials with attractive physicochemical properties for applications in medicine, food, agriculture, and environment. The mechanisms presented here help to understand the cellular and molecular events in microbes toward metal adsorption, intake, accumulation, nanoparticle synthesis and stabilization. In view of the background, this Special Issue will enable researchers and bioengineers to optimize the nanoscale processes of the microscale organisms for developing tailor made nanostructures with desired morphological features and applications.

## Author Contributions

SG and RJT drafted, corrected, and approved the editorial note.

## Funding

RJT recognizes National Sciences Engineering Research Council (NSERC) of Canada for a Discovery grant. SG acknowledges the Department of Science and Technology (DST), Ministry of Science and Technology, Government of India and Jawaharlal Nehru Center for Advanced Scientific Research, India for funding under the Post-doctoral Overseas Fellowship in Nano Science and Technology [Ref. JNC/AO/A.0610.1(4) 2019-2260].

## Conflict of Interest

The authors declare that the research was conducted in the absence of any commercial or financial relationships that could be construed as a potential conflict of interest.

## Publisher's Note

All claims expressed in this article are solely those of the authors and do not necessarily represent those of their affiliated organizations, or those of the publisher, the editors and the reviewers. Any product that may be evaluated in this article, or claim that may be made by its manufacturer, is not guaranteed or endorsed by the publisher.
